# The Impact of Suicide Utility Perception on News over Terminally Ill Patients’ Suicide Attitudes: A Pilot Study

**DOI:** 10.3390/ijerph18168784

**Published:** 2021-08-20

**Authors:** Diego Garcia-Fernández, Samuel Fernández-Salinero, Gabriele Giorgi, Gabriela Topa, Ana María Marcos Del Cano

**Affiliations:** 1 International School of Doctorate, The National Distance University (UNED), 28040 Madrid, Spain; 2.diego.garcia@gmail.com; 2 Psychology Department, Universidad Rey Juan Carlos, 28040 Madrid, Spain; samuel.fernandez@urjc.es; 3 Department of Human Science, European University of Rome, 00163 Rome, Italy; gabriele.giorgi@unier.it; 4 Social and Organizational Psychology Department, The National Distance University (UNED), 28040 Madrid, Spain; 5 Faculty of Law, The National Distance University (UNED), 28040 Madrid, Spain; amarcos@der.uned.es

**Keywords:** suicide, suicidal ideation, suicide risk factors, depressive symptomatology, terminally ill people’s euthanasia, euthanasia

## Abstract

Suicide represents a very important issue in public health. For approaching attitudes toward suicide, we have developed an instrument that, following previous recommendations, assesses specific thoughts related to the perception of suicide utility in the press. First of all, we will test the psychometric properties of the scale we created ad hoc for assessing suicide utility perception. After that, we expect to find that the suicide utility perception in the press will have a statistically significant impact on positive attitudes toward terminally ill patients’ suicide (Hypothesis 1). In addition, this relationship will be mediated by suicide legitimation (Hypothesis 2). This mediation will be moderated by depressive symptomatology (Hypothesis 3). The sample was composed of 66 Spanish participants. Suicide legitimation was significantly related to the positive evaluation of terminally ill people’s euthanasia. Finally, when the levels of depression’s psychological concomitants increased, the support for terminally ill people’s euthanasia increased as well. Implications and limitations have been discussed.

## 1. Introduction

Suicide represents a very important issue in public health. Our research departs from previous research recommendations [1], and we tried to develop an instrument to achieve a better understanding about the attitudes towards suicide.It is estimated that in 2015 there were about 788,000 deaths caused by suicide [2]. In Europe, previous research has shown that suicide rates range from 30.8 per 100,000 inhabitants to 2.2. One of the problems around suicide is that few identified antecedents can help the prevention, as well as its relationships with other health-related problems, such as alcohol consumption [3].

Specifically, suicide is the main non-natural death cause in Spain [4]. It overcame traffic accidents as the main non-natural death cause in 2007 and is committed more frequently by men. However, recently women’s suicide has risen as well. One of the main difficulties around tackling suicide is that it is frequently silenced [5]. Among the efforts invested in prevention and the early detection of this problem, there have been different risk factors identified. Particularly in teenagers [6], risk factors such as adverse events, sexual and psychological abuse, psychiatric problems, impulsivity, parental suicide-bereavement, and hopelessness have been found [7,8].

In this line, recent studies have stated the need for more and wide research that helps to shed light and understand this public health problem [2]. In this connection, recent research has included mass media analysis, finding that there was a relationship between internet search topics and suicide [5]. Moreover, other research has presented predictive models of suicide based on internet searches [9]. In this line, other researchers have found similar results [10,11,12]. This is in line with previous research that highlights the influence of media reporting of celebrity suicides, which tends to increase suicide rates [13]. Due to the multicausality of the phenomenon, recent research has pointed that suicide and suicidal behavior is a challenge for clinical, social, and theoretical fields [13]. Our research is based upon previous research recommendations that highlighted the necessity of continuing exploring and developing ways to prevent and tackle suicide.

It has been studied that press and mass media campaigns are increasingly becoming an important factor in preventing suicide [14]. In the 1960s, debate began around the relationships between media portrayals of suicide and subsequent suicidal behaviors [15]. Results of this research showed that the newspaper suicide blackouts had ambiguous results. For example, some authors [15] reported that in the seventies, media suicide blackouts showed from no impact on suicide rates to a significant lowering of the rates on females. After more than 40 years of research in the field, it seems legitimate to affirm that there is a relationship between newspaper reports of suicide and suicides. Following Pirkis’s research [15], much is still not known about media and suicide and, furthermore, mass media are changing into a digital format. There is a scarcity of research in the field of news and attitudes, and our research aims to shed light on the relationship between these two variables.

Mass media is an important factor in changing attitudes and behaviors through the passive reception of information [14], and some research brings to light that suicides must be reported responsibly. Surprisingly, it has been found that mass media campaigns have little to no impact on suicide or suicidal behavior [16]. Based on previous research we can pose that suicide reporting in the press is related to suicide behavior, and suicide prevention campaigns do not show an impact on suicide levels.

Specifically, evidence has been found that relates suicide-related internet activity and some aspects of suicidal behavior. This relationship has been found strongly and consistently [17]. The conclusions of previous research propose that suicide in news and information media may influence copycat behavior in particular circumstances [17]. Hence, individuals may perceive suicide as a useful way of coping.

Since mass media enhance identification with authors and decrease stigma, it may sensitize individuals to understand certain situations and normalize suicide in some cases. Suicide in terminally ill patients is a highly controversial topic currently [18] and some research has shown that above 60% of terminally ill patients support this practice. In addition, a wide section of the literature tends to focus on understanding the opinions of physicians about terminally ill patients. Some suicide research does not call for a censure of suicide in media but appeals to responsibility in the media’s presentation.

Due to this ambiguity, it is difficult to evaluate how to approach suicide in the press. Since suicide is a very controversial topic, it is very difficult to assess attitudes towards it [1]. To solve this trouble, and for a better understanding of the phenomenon, we have developed an instrument following the previous recommendation for the evaluation of the perception of suicide utility in press [1].

In this line, it has been shown that suicide is a very complex attitudinal phenomenon. Individuals appear to show different feelings, cognitions, and behaviors if suicide is committed by themselves, people in general, or people near to them [19]. Based on previous research [20], people may have a general vision about suicide legitimation. Due to this, it is necessary to understand certain cases of suicide, such as terminally ill patients. Based on the abovementioned research, we hypothesize that the attitudes towards suicide as a resource may impact the attitudes towards suicide in general, or even suicide itself. This is in line with studies that state that there is evidence that individuals with a more accepting view of suicide tend to show higher levels of suicidal ideation and have attempted suicide in the past [21].

In suicide research, we have found that instruments are usually designed for patients or physicians, finding a lack of research on general population attitudes. Since news may reach worldwide, our research tries to fill that gap and offers an approximation to suicide attitude measurement on the general population. This may help to shed light upon the information processing carried out by readers. Our study shall be understood as a pilot study and the starting point of future research in the field.

There is a huge debate about the legalization of terminally ill patients’ suicide, and there are different opinions among the general public, physicians, and patients [22,23]. Previous research found that the general population and patients tend to agree with assisted suicide, while most physicians tend to oppose it [22]. Since there is polarization on attitudes over this topic, social mass media plays a central role in these processes.

Trying to understand suicide complexity, there is a considerable amount of investigation that relates depression and suicidal behavior [24]. Recent research has related depression and external symptoms such as substance misuse, risk-taking, and difficulties in impulse control [25]. As depression comprises cognitive factors that may affect attitudes, it is interesting to understand how people with depressive symptomatology may respond to the perception of suicide utility in news and its attitudes toward suicide in terminally ill patients.

Having said this, and recognizing the lack of investigation in this field, the main objective of this paper is to analyze the influence of the suicide utility perception in news over the attitudes towards suicide in terminally ill patients. This relationship will be mediated by suicide legitimation and moderated by cognitive depression symptoms.

Specifically, one of the main objectives of this research is to analyze if the exposure to news that helps to understand suicide is useful or tends to increase the attitudes towards suicide in terminally ill patients.

First, we will test the psychometric properties of the scale we created ad hoc for assessing suicide utility perception. After that, we expect to find that the suicide utility perception in the press will have a statistically significant impact on positive attitudes toward terminally ill patients’ suicide (Hypothesis 1). Additionally, this relationship will be mediated by suicide legitimation (Hypothesis 2). This mediation will be moderated by depressive symptomatology (Hypothesis 3).

## 2. Method

The current study follows a quantitative correlational transversal design. Due to the pilot character of our research, our sample was selected by incidental nonrandomized parameters. Sample characteristics may be seen in Table 1. The sample was composed of 66 Spanish participants. Data were collected online. We ensured confidentiality and anonymity of every participant, and they gave informed consent to voluntarily participate in our research. Related to the composition of our sample, 39.4% (26 subjects) of the sample were men while 60.6 % were women (40 subjects). The mean age was 41.88 (SD = 11.18), and the minimum participant’s age was 23 years old. Related to academic level, most of our sample had a university degree (80.3%; 53 individuals), only one subject had basic studies (1.5%), four subjects had vocational training (6.1%), and four subjects finished baccalaureate (6.1%). Further, four more subjects decided not to respond (6.1%). Concerning the contract type, 66.7% (44 subjects) of the sample were employees, while 8.1% (9.1%) were freelancers. Of the remainder, 13.6% (9 subjects) were students and there was only one retiree (1.5%). Lastly, related to the frequency of newspaper reading, 35 subjects (53%) read the newspapers every day. 19 subjects (28.8%) reported reading the newspapers more than three times per week, 6 subjects (9.1%) responded that they read the newspapers less than three times a week, and another 6 subjects (9.1%) reported that they read the press rarely.

### 2.1. Instruments

#### 2.1.1. Suicide Utility Perception Rating

In order to assess this variable, we developed an ad-hoc instrument. The main aim of this instrument was to assess the perception of the social utility of reading about suicide. The instrument was composed of 17 items. The exploratory factorial analysis showed 4 different factors: social utility, conscientization, help utility, and warn utility. Each item was rated in a 5-point Likert type scale from 1 “strongly disagree” to 5” strongly agree”. After conducting confirmatory factor analysis, 10 items were included. Cronbach alpha values yielded 0.85 for the overall scale. Examples of the items composing this scale were: “Some readers may feel identified with the situation of a person who committed suicide after reading in the press”, “Reading about suicide in the press makes me think that is something that may happen to anyone”.

#### 2.1.2. Legitimacy of Suicide and Suicide of Terminally Ill Patients

For the measurement of these variables, the Attitudinal Beliefs Questionnaire about Suicidal Behavior (CCCS-18) was used. This original questionnaire was created in Spain [20]. This questionnaire has demonstrated its utility in predicting suicidal tendency [26]. The original instrument was composed of an 18-item Likert-type scale. Response scale was pointed from 1 = “Strongly disagree” to 7 = “Strongly agree”. Legitimacy of suicide factor was composed of 5 items, and its Cronbach’s alpha value was = 0.84. It measures the perception of suicide as a logically acceptable act. An example of this factor is “Suicide should be a legitimate way of dying”. On the other hand, the suicide of terminally ill patients was composed of 4 items with Cronbach’s alpha = 0.82. It measures the individual’s support for terminally ill patients’ suicide. An example of this factor is “Dignified suicide should be permitted to those suffering from incurable diseases”. In our sample, Legitimacy of suicide showed a Cronbach’s alpha = 0.85 and suicide of terminally ill patients yielded a Cronbach’s alpha = 0.90. Despite that some researchers have argued that they measure the same factor [27], we will test the factors separately in a mediation model.

#### 2.1.3. Cognitive Depression Content

For the assessment of this variable, we used the Zung self-rating depression scale [27] (Z-SRDS). It is intended to map the complex behavioral, cognitive, and affective concomitants of depression. It has been demonstrated to be an excellent option to address depression [28]. For our purposes, we used the cognitive factor of this instrument, which is composed of 10 Likert-type items, from 1 “Strongly disagree” to 7 “Strongly agree,” and rate the thought content of depression. The original scale’s Cronbach alpha ranged from 0.88 to 0.93. Our sample showed a Cronbach’s alpha = 0.89. An example of the items is “I still enjoy the things I used to do”.

### 2.2. Procedure

The Ethics Committee of the National Distance Education University approved the study protocol in accordance with the Declaration of Helsinki (protocol number 180717). In the present study, potential participants were informed of the aims of the research being carried out and the conditions regarding anonymity and the voluntary nature of their collaboration. They were also assured they could withdraw from the study at any time without penalty. The questionnaire was sent to the participants through the Google Forms application. Before starting to fill the questionnaire, informed consent must be accepted.

After the data collection, we conducted an exploratory factor analysis (EFA) with varimax rotation. This procedure helped us to assess the reliability of our ad-hoc scale. First, we assessed the factorial structure of our instrument and checked the Cronbach’s alpha indicator. Thereupon, we conducted a confirmatory factor analysis (CFA) to test the fit of our model and guarantee the usability of the scale. We used the split-half method. According to Awang et al.’s guidelines [29], we used the following indexes: Chi-square/df, Comparative fix index (CFI), Goodness of fit index (GFI), and normed fit index (NFI). We also included the root mean square error of approximation (RMSEA). These abovementioned indexes guaranteed that our model fixes the data.

Data analyses were conducted by IBM SPSS v. 24 (IBM Corp., Armonk, NY, USA) and IBM AMOS v.23 (IBM SPSS, Chicago, IL, USA).

## 3. Results

### 3.1. Exploratory Factor Analysis

The first step that we followed was to evaluate the factorial structure of the questionnaire. For this purpose, we conducted a Kaiser-Mayer-Olkin (KMO) test, which showed adequate values for conducting the factorial analysis (0.73). Moreover, we checked Bartlett’s Sphericity test, which showed adequate values as well (χ^2^ (136) −543.83, *p* < 0.001).

The principal axis factoring procedure showed a five-factor structure. We used as criteria the eigenvalues over 1.00, and factor loadings above 0.30. Taking these criteria into account we preferred to extract a four-factor matrix because the fifth factor was composed only of one item. The factor loadings may be seen in Table 2.

### 3.2. Confirmatory Factor Analysis

Having explored the factor structure of our instrument, we conducted a confirmatory factor analysis in order to find the best model to use in our research. First, we conducted an analysis with the 17 items, and we adjusted the model. We used the criteria suggested by Awang [29]; chi-square/df should be below 2. CFI, GFI, and NFI should be above 0.90 for a good fit, and RMSEA should be below = 0.08. We eliminated the items with lower factor loadings in the confirmatory analysis. Results of the two purposed models can be seen in Table 3.

**Figure 1:**
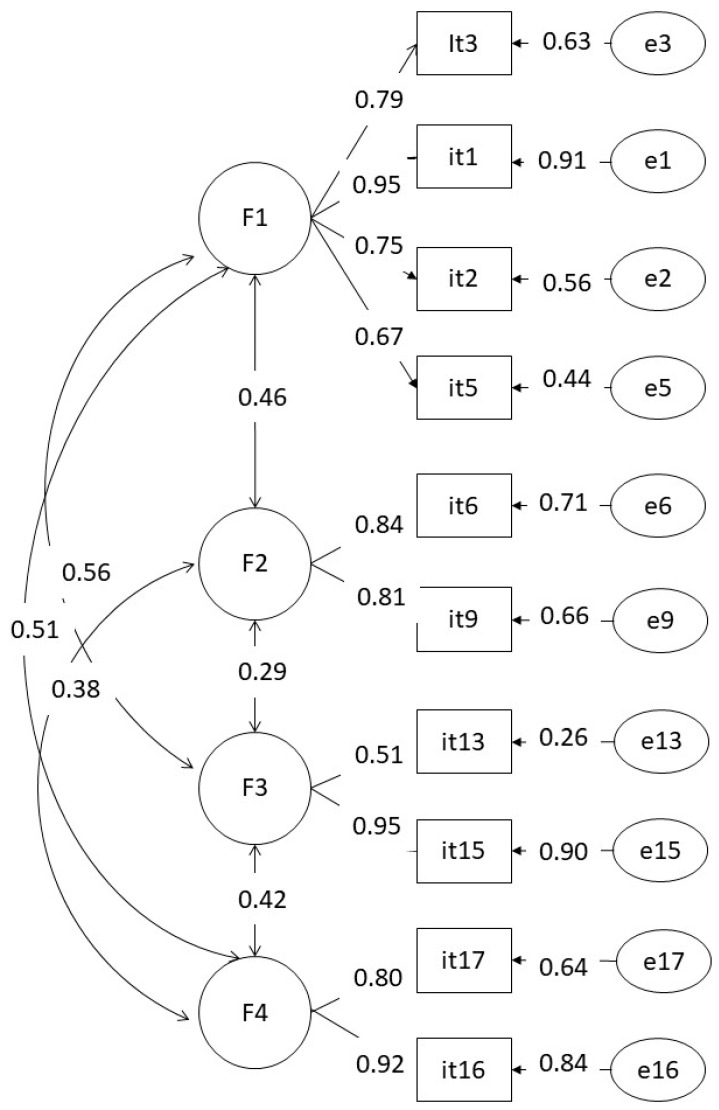
Path diagram and estimates for the four factors model suggested for this instrument.

As can be seen, the 10-item model offers a better adjustment to the data. The statistical diagram may be seen in Figure 1.

Having analyzed our model, it yields good enough indexes for using it in our research, guaranteeing its reliability. Cronbach’s alpha remained 0.85.

For testing our hypotheses, we conducted a moderated mediation model developed by Hayes [30]. Concretely, we utilized model 7 of his PROCESS macro for SPSS. The first step for conducting a moderated mediation analysis is to weight the correlation between variables. Age, gender, academic level, and press reading frequency did not show any significant relationships. Due to this, they were not included in the final model. The correlation matrix may be seen in Table 4.

The PROCESS macro is based on a bootstrapping procedure that extracts 1.000 random samples for assessing the hypotheses. For a better explanation, we will expose direct relationships, simple mediation, a simple moderation model, and mediated moderation indexes.

### 3.3. Direct Relations and Simple Mediation

The first step of our model is to assess the influence of the suicide utility perception over the positive evaluation of terminally ill people euthanasia. We found that this direct relationship was statistically significant (B = 0.72; SE = 0.17; 95% CI (0.36; 1.07), *p* < 0.001. As such, we have enough evidence to accept hypothesis one. In order to continue with our model, we will test other direct relations. Suicide legitimation was significantly related to the positive evaluation of terminally ill people euthanasia (B = 0.68; SE = 0.08; 95% CI (0.53; 0.84), *p* < 0.001). When evaluating the influence of our variables on suicide legitimation, we found that neither the suicide utility perception (B = − 0.50; SE = 0.79; 95% CI (−2.10; 1.09), *p* > 0.05), nor the depression psychological concomitants (B = −2.52; SE = 1.73; 95% CI (−5.99; 0.95), *p* > 0.05) were statistically significant. However, we found that the simple mediation (utility perception -> legitimation -> terminally ill people euthanasia) model was supported by our data (B = 0.58; SE = 0.19; 95% CI (0.19; 0.97)). Having said this, we have enough evidence to confirm hypothesis 2.

### 3.4. Moderation Analysis

The simple moderation analysis (utility perception impacts on suicide legitimation, being these relationships moderated by depression psychological concomitants) showed not statistically significant values (B = 0.78; SE = 0.45; 95% CI (−0.13; 1.69), *p* > 0.05). However, we found that at 16th percentile of depression psychological concomitants, effects were not significant (B = 0.42; SE = 0.33; 95% CI (−0.23; 1.09), *p* > 0.05), there were at 50th (B = 0.97; SE = 0.28; 95% CI (0.41; 1.53), *p* < 0.01) and 84th (B = 1.52; SE = 0.49; 95% CI (0.52; 2.51), *p* < 0.01) percentile.

### 3.5. Moderated Mediation Analysis

**Figure 2:**
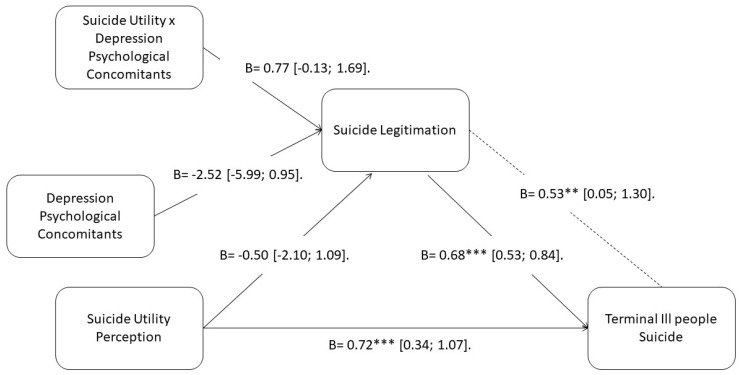
Statistical model where coefficients and effect sizes may be observed. Note: *** *p* < 0.001. ** *p* < 0.01.

Lastly, we assessed the final model and confirmed it was statistically significant. Utility perception (X) was related to terminally ill subjects’ euthanasia (Y), through suicide legitimation (M), being these relationships mediated by depression psychological concomitants (W) (B = 0.53; SE = 0.31; 95% CI (0.05; 1.30)). When evaluating the conditional effects of our model we found that at lower levels of depression psychological concomitants, support for terminally ill people’s euthanasia was not significant (16th percentile; B = 0.29; SE = 0.28; 95% CI (−0.39; 0.77)). Nevertheless, while increasing the levels of depression psychological concomitants, the support for terminally ill people’s euthanasia increased as well (84th percentile; B = 1.04; SE = 0.29; 95% CI (0.51; 1.67)). Because of this, we have enough evidence to confirm hypothesis 3. A complete statistical model may be seen in Figure 2.

## 4. Discussion

The main objective of this paper is to analyze the influence of suicide utility perception in news over the attitudes towards suicide in terminally ill patients. This relationship will be mediated by suicide legitimation and moderated by cognitive depression symptoms.

Since suicide represents a very important issue in public health, representing the main non-natural cause of death in Spain [4], we found that it is necessary to address some interrogatives highlighted by previous research [13]. Recent research has stated that being exposed to suicide news is related to copycat conduct and higher rates of suicide [5]. Based upon this association, our research tries to find out the cognitive mechanisms that help to link suicide exposure and attitude change. Since previous research recommends continuing to explore the mechanisms around suicide, our study fills the gap, focusing on the process of subjects’ change of attitudes when exposed to suicide news.

Here is where our research represents a new field of study. Our research highlights that the perception of the utility of suicide in news is directly and significantly related to the attitudes toward terminally ill patients’ suicide. It seems that there is a change of attitudes towards the perception of suicide. This is in line with previous research that shows that suicide-related topics in the newspaper are related to a higher suicide rate. The new aspect of our research is to discover that reading about the utility of suicide does not increase the legitimation of suicide, but the approbation of terminally ill patients. There is not a general change of attitudes towards suicide, but future research should explore if this change of attitudes is related to empathic processes. Previous research has shown that suicide is a very complex attitudinal object and must be investigated deeply [20]. It would be interesting to test if the perception of suicide utility on the press may impact one’s own suicidal tendencies. Since it is a very complex and ethically compromised topic, future research should find a way to evaluate how news impacts the different types of suicide. Moreover, since suicide antecedents are not so clear and are frequently silenced [5], our research represents a new contribution to this field. First, our research draws from previous papers where it has been said that psychological depressive symptoms are related to suicide [8], but the innovation is that in our research these symptoms act as a moderator. Specifically, psychological depression symptoms did not show any relationship to the attitudes toward terminally ill patients, but a moderation effect. It is interesting that, in our paper, depressive concomitants are not related to suicide legitimation, even when are combined with suicide utility perception. However, when increasing the depression levels, from the 50th percentile our data showed that there is a significant relationship with terminally ill patients through suicide legitimation. Future research should explore deeper these relationships to prevent suicidal attitudes in depressive subjects. This is in line with previous research that strongly relates depression and suicide [31].

Recent research has shown that mass media campaigns are an important factor in preventing suicide [14], while others have shown no effect to this end [17]. Our research contributes to understanding why it may happen that some of them have not succeeded. It seems that there are dispositional variables (such as depression) and environmental variables (such as the circumstances around suicide) that might have an impact on the attitudes towards suicide. Understanding these circumstances may enhance some favorable attitudes towards certain cases of suicide, specifically in people with depressive symptomatology. Our research matches with previous research that recommends addressing suicide cautiously and respectfully.

In line with previous research, our study finds that there is a direct relationship between the exposure to news and positive attitudes towards terminally ill patients. It seems that, based upon the findings of previous research that have found ambiguous relationships [16], it is needed to specify the conditions when being exposed to suicide news can lead to attitudinal change. Attitudes towards suicide are a very important topic in preventing suicide. Since suicide attitude is influenced by culture, it is important to study. As previous research recommends, understanding attitudes towards suicide may be useful in suicide prevention or in providing interventions. This is in line with studies that state that there is evidence that individuals with a more accepting view of suicide tend to show a higher level of suicidal ideation and have attempted suicide in the past [22].

Moreover, our research confirms that social media plays a very important role in attitudinal change. Based upon research that highlights that there is a division between physicians and the general population around suicide [23], future research should investigate if exposure to suicide news has the same impact in these two different populations.

## 5. Limitations

Our paper has some important limitations. First, being a pilot study, the sample is not representative and is too small. This may affect the generalization of the results. Future research should extend the sample and randomize the experiment to gain validity. Secondly, the lack of established measures to assess the main variables of our study is a major limitation. Hence, we created the instrument and validated it in the same study. We believe that future research should apply validated instruments, or test the reliability of our instrument. Thirdly, future research should investigate if our findings are similar in different populations, such as physicians and the general population. In addition, there may be some differences in clinical or subclinical populations. Longitudinal studies are required to test the influence of press reading over the attitudes towards suicide over time.

Finally, our research has shown that suicide patterns do not show a significant impact on terminally ill patients’ euthanasia or suicide legitimation. However, in subjects with higher values of this variable, it seemed to appear a significant moderation of the influence of perception of utility over terminally ill patients’ suicide, through suicide legitimation. It would be interesting to replicate our research in a clinical population to test the strength of this association.

We have shown that the perception of utility in news has an important impact on suicide attitudes and news editors should be aware of this peculiarity. It is required to test the impact of this utility on suicidal tendencies. Being aware of these aspects, we could be a more responsible society, and develop models that may tackle the huge impact that suicide has all over the world.

## 6. Conclusions

The main objective of our research is to analyze the influence of the suicide utility perception in news over the attitudes towards suicide in terminally ill patients. We stated that this relationship would be mediated by suicide legitimation and moderated by cognitive depression concomitants. Our findings confirm that there is a direct and statistically significant relationship between the suicide utility perception in news and attitudes towards suicide in terminally ill patients. In addition, we found that depression equivalent concomitants were direct and significantly related to attitudes towards suicide in terminally ill patients. However, we did not find that depression was related to suicide legitimation or suicide utility perception.

We found that depression moderated the relationship between suicide utility perception and suicide legitimation. This moderation was only significant from the 50th percentile and above. When evaluating mediated moderation, we found that our model was significant from the 50th percentile and above of depression equivalents. Future research should clarify these relationships. Due to the pilot nature of our study and the sampling limitations, our results may be not generalizable.

## Figures and Tables

**Table 1:** Sociodemographic data and percentages from our sample.

Variable	Frequency	Percentage
Gender:		
Men	26	39.4%
Women	40	60.6%
Age M = 41.88; SD = 11.18		
Academic level:		
University degree	53	80.3%
Basic studies	1	1.5%
Vocational training	4	6.1%
Baccalaureate	4	6.1%
Frequency of reading		
Everyday	35	53%
More than three times per week	19	28.8%
Less than three times per week	6	9.1%
Rarely	6	9.1%

**Table 2:** Exploratory Factor Analysis rotated factor loadings.

Item	Social	Awareness	Help	Warn
Item 1	0.85	0.01	0.15	0.28
Item 2	0.77	0.08	0.02	0.19
Item 3	0.76	0.17	0.07	0.20
Item 4	0.66	0.46	−0.03	−0.12
Item 5	0.62	0.31	0.38	0.09
Item 6	0.37	0.75	0.00	0.17
Item 7	0.04	0.68	−0.12	−0.10
Item 8	−0.15	0.67	0.34	0.04
Item 9	0.28	0.67	0.12	0.26
Item 10	0.10	0.66	−0.21	0.24
Item 11	0.46	0.52	0.04	−0.23
Item 12	−0.17	−0.11	0.76	0.00
Item 13	0.24	−0.03	0.72	0.05
Item 14	0.29	0.38	0.58	0.05
Item 15	0.45	−0.10	0.52	0.32
Item 16	0.28	0.06	0.02	0.87
Item 17	0.13	0.15	0.13	0.86

Note: Cronbach’s alpha value showed 0.86, and the four factors structure explained 64.5% of the variance.

**Table 3:** Fit indexes for Confirmatory Factor Analyses.

Model	*χ*^2^/df	*χ* ^2^	CFI	NFI	RMSEA
17 items	1.76	0.00	0.82	0.68	0.10
10 items	1.01	0.44	0.99	0.90	0.01

Note: CFI = Comparative Fix Index; NFI = Normal Fit Index; RMSEA = Root-mean-square error of approximation.

**Table 4:** Pearson Correlation Matrix.

	M	SD	1	2	3
Utility	1.89	0.66	-	-	-
Legitimation	3.50	1.63	0.48 **	-	-
Psychological	5.17	1.66	0.12	0.22	-
Terminal	3.60	0.71	0.61 **	0.77 **	0.27 *

Note: ** *p* < 0.01. * *p* < 0.05. M: Mean; SD = Standard Deviation.
